# Co-Administration of Curcumin and Bromocriptine Nano-liposomes for Induction of Apoptosis in Lung Cancer Cells

**DOI:** 10.29252/ibj.24.1.24

**Published:** 2019-08-28

**Authors:** Mojgan Sheikhpour, Majid Sadeghizadeh, Fatemeh Yazdian, Ali Mansoori, Hassan Asadi, Abolfazl Movafagh, Seyed Sadegh Shahraeini

**Affiliations:** 1 Department of Mycobacteriology and Pulmonary Research, Pasteur Institute of Iran, Tehran, Iran;; 2 Microbiology Research Center (MRC), Pasteur Institute of Iran, Tehran, Iran;; 3 Department of Genetics, Faculty of Biological Sciences, Tarbiat Modares University, Tehran, Iran;; 4 Department of Life Science Engineering, Faculty of New Sciences and Technologies, University of Tehran, Tehran, Iran;; 5 Department of Medical Genetics, Cancer Research Center, Shohada Hospital School of Medicine, Shahid Beheshti University of Medical Science, Tehran, Iran

**Keywords:** Apoptosis, Bromocriptine, Curcumin

## Abstract

**Background:**

In recent years, nanotechnology with modern advances in the macromolecular design of nano-carriers has proved to be helpful in the development of drugs delivery systems. This research represents a novel co-administration of nano-vehicles, known as liposomes. Liposomes efficiently encapsulate curcumin and BR in a polymer structure, which results in enhanced aqueous solubility of the mentioned hydrophobic agents and higher bioavailability of the drugs.

**Methods:**

Preparation of curcumin and BR liposomes were carried out by the thin film method, and the amounts of purified drug and its release were analyzed. After dose determination, the human lung cancer cells (QU-DB) were exposed to BR and curcumin liposomes for 12, 24, and 48 h. Then the viability and apoptosis assays were carried out by using MTT and flow cytometry technique, respectively.

**Results:**

In this research, *in vitro* anti-cancer effects of former nano-formulations on lung cancer cells was confirmed, and no cytotoxicity effects of these nano-preparations were observed in the normal cells (HFLF-PI5).

**Conclusion:**

Our findings suggest the nano-liposomal drugs as effective anti-cancer agents; however, additional clinical examinations are required.

## INTRODUCTION

The most common cancer in the world is lung cancer^[^^1^^,^^2^^]^. Despite numerous developments in surgery, chemotherapy, and radiotherapy over the past decades, the fatality rate of lung cancer has still continued largely unaffected, which is mostly because of its metastasis^[^^3^^]^. New treatment approaches for lung cancer patients are highly demanded because of the overall poor prognosis. Meanwhile, due to the low bioavailability of curcumin, its biological activity is severely limited, though the curcumin therapeutic index is promising^[^^4^^]^. 

For handling drug delivery issues, the most important nanoparticle systems are liposomes, polymer conjugates, polymeric micelles, dendrimers, nano-shells, proteins, and nucleic acid-based nanoparticles. Among these tools, liposomes and polymer-based nano-formulations create a common nanoparticle therapeutic agent, available for the clinical use^[^^5^^]^. Liposomes  are artificially constructed vesicles consisting of a phospholipid bilayer and have the ability to adjust the bio-distribution of drugs^[^^6^^]^. The current trials for various liposome constructions and a large number of commercially presented therapeutics seem to be promising^[^^7^^]^. The hydrophobic properties of curcumin has made it as a good candidate for liposome integration and encapsulation in the lipid layer of the liposomes^[^^8^^]^. 

Studies have suggested that the integration of curcumin into liposomes significantly augmentes its bio-availability. Moreover, the activity of liposomal curcumin is more favorable than that of curcumin alone^[^^9^^]^. A primary therapeutic drug for adenomas is BR. BR binds to the dopamine D2 receptors on pituitary epithelial cells to prevent prolactin secretion. Nowadays, BR is used to treat various diseases such as Parkinson, acromegaly, addiction, hyperprolactinemia, high growth hormone-secreting disorders, cyclical mastalgia, and type 2 diabetes^[^^6^^]^. BR stimulates dopamine receptors and has anti-mitotic and anti-tumor properties. D2 receptor mRNA has been identified in all BR-sensitive tumors^[^^5^^,^^10^^]^. The results of a previous research showed that significant changes occur in the expression of D2-like dopamine gene receptor in NSCLC^[^^11^^]^.

In the present research, *in vitro* apoptosis occurrence by BR was examined in a lung adenocarcinoma cell line. The primary goal of this study was to estimate the possible curcumin and BR partitioning into the liposomes and adjustment of curcumin and BR encapsulation in liposomes. Also, we tried to assess the effect of curcumin-loaded and BR-loaded liposomes on lung cancer cells. To achieve this goal, DLS examination, anti-proliferative effects study using a MTT-based assay, and flow cytometry were applied. 

## MATERIALS AND METHODS

**Chemicals and cells**

 Curcumin and BR were obtained as a gift from Genetics Department of Tarbiat Modares University (Tehran, Iran). Soybean phospholipids with 75% phosphatidyl-choline, 2-distearoyl-sn-glycero-3-phosphoethanol-amine, and sodium salt (DSPE-mPEG-2000) were procured from Lipoid GmbH (Ludwigshafen, Germany). Cholesterol was purchased from Sigma-Aldrich (St. Louis, MO, USA). All other chemicals and solvents used in this investigation were of analytical grade. Human lung carcinoma cell line, QU_DB, and human lung cell line, HFLF-PI5, (as a control) were provided from the National Cell Bank of Pasteur Institute of Iran, Tehran.

**Preparation and characterization**

Curcumin and BR liposomes were prepared by the thin film hydration-sonication method. Hydration was performed with 1300 μL deionized water at 65 °C for 60 minutes, using a rotary instrument (Heidolph, Germany). The resulting vesicles were then sonicated to reduce the mean size. The size distribution and polydispersity index of the drug-loaded liposome were evaluated using the DLS technique. All the measurements were carried out in triplicates at room temperature, and their mean value was reported. 

**Drug entrapment efficiency**

Free unentrapped drugs were separated from drug-loaded liposomes using the dialysis membrane (cut off: 12-14 kDa). After digesting the liposomal vesicles with isopropanol (99% purity), the amounts of liposomal drug entrapped were analyzed by a UV spectrophoto-meter at 422 nm^[^^12^^]^. 

**Release assay**

The release of drug from liposomes against PBS was monitored by dialysis at pH 7 at 37 °C for 96 hours. The calculation of drug release was performed at different times in the PBS solution. Further, drug release calculation was conducted using a calibration curve by a UV spectrophotometer.

**MTT assay**

The cell proliferation effects of BR and curcumin liposomes were examined by the MTT assay^[^^11^^,^^13^^]^. The cells were seeded at 10^4^ cells/well (0.1 ml) in triplicates in a 96-well plate and treated with varying doses of BR (12.5-25 µM) and curcumin liposomes (12-20 µm), after 12-, 24-, and 48-h incubation at 37 °C. 

**Flow cytometry**

The QU-DB cells were treated with different concentrations of BR (12.5-25 µM) and curcumin liposomes (12-20 µm) for 12, 24, and 48 h, and then Annexin-V-Fluos staining kit (Roche, Germany) was used for detection of apoptosis^[14]^. The treated cell pellets were resuspended in 100 μl of Annexin-V-Fluos solution, and after 15-min incubation at 25 °C, they were analyzed on a FACSCalibur machine (BD Biosciences, CA, USA). 

## RESULTS

**Nanoparticle** **size**

**Fig. 1:**
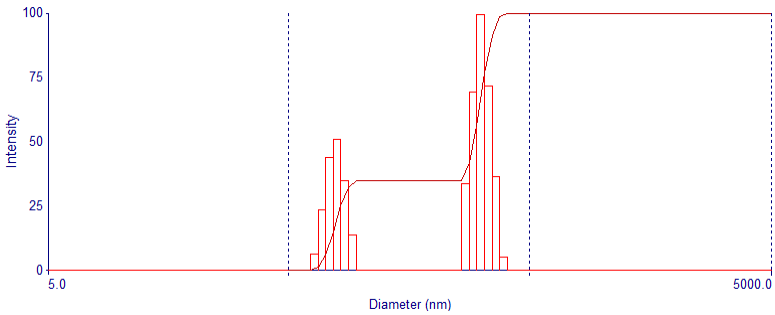
DLS of liposome-curcumin nanoparticles

**Fig. 2:**
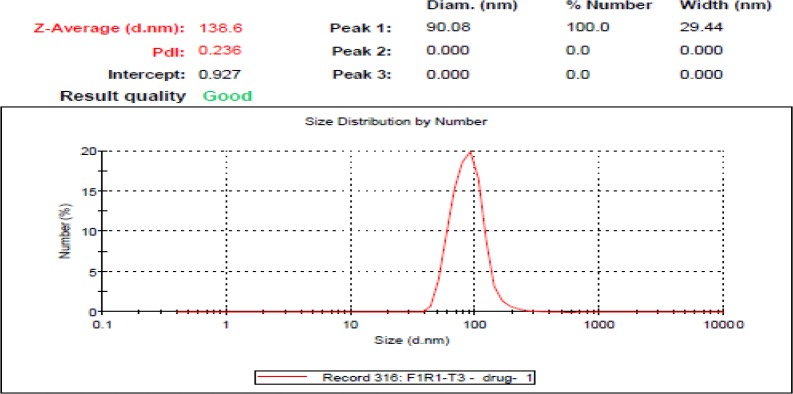
The intensity of liposome-BR

The DLS of liposome-curcumin is presented in Figure 1. Around 20% of the particles are in the range of 100 nm. More analysis indicating that the remaining particles were about 80 nm in size. Figure 2 illustrates the intensity of liposome-BR, where the diameters of nanoparticles are less than 50 nm. 

**Drug release assay**

**Fig. 3:**
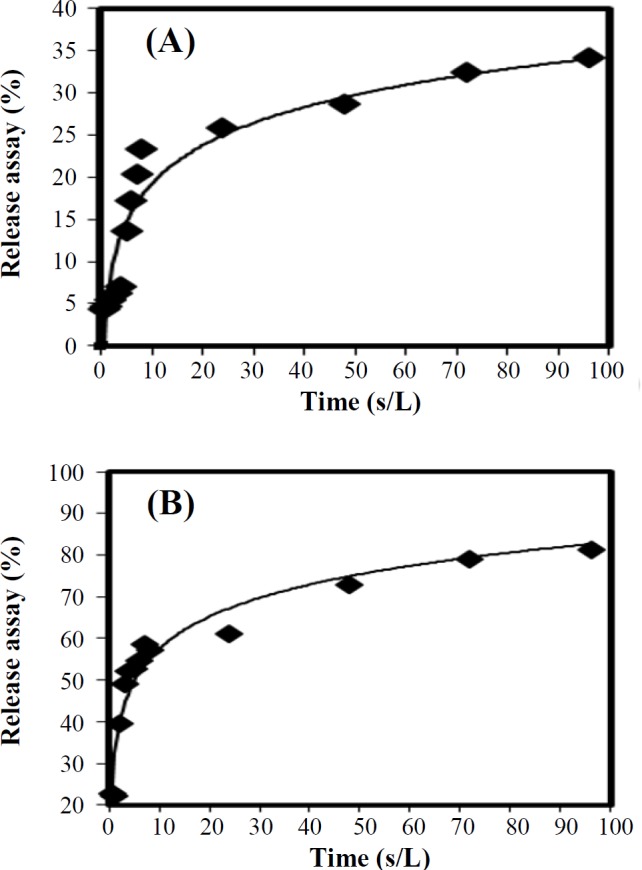
Release of liposome-curcumin (A) and liposome-BR (B) nanoparticles

Figure 3 demonstrate the rate of drug release from liposomes. As revealed in the Figure, the extent of drug release of liposome-BR nanoparticles is greater than that of liposome-curcumin nanoparticles at the same time. In particular, during 10 h, 60% of the liposome-BR nanoparticles and only 20% of the liposome-curcumin nanoparticles demonstrated the drug release.

**Drug entrapment efficiency and stability test**

Entrapment efficiencies were calculated as 90.89% and 80.41% for the liposome-curcumin and liposome-BR nanoparticles, respectively. Also, the stability of the liposomal suspensions was evaluated after three months of storage at room temperature at 4 °C. The particle size supply, morphology, and drug encapsulation efficiencies of the samples were determined as a function of the storage time. The results indicated that only 15% of the liposomal suspensions efficacy was diminished.

**The effect of prepared liposomes on cell viability**

**Fig. 4:**
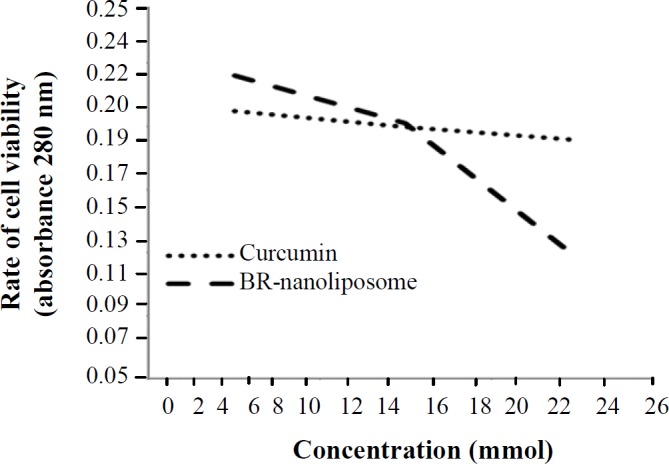
Effect of various doses of curcumin and BR nano-liposomes on QU-DB cells

Different concentrations of BR (12.5-25 µM) and curcumin (12-20 µM) liposomes were applied on the QU-DB cells in triplicate for 12, 24, and 48 h, and MTT assay was performed after the treatment. According to Figure 4, the proliferation of cancer cells reduced significantly at the concentrations of 20 to 25 µM for BR and at 16 to 20 µM for curcumin. In addition, the results of our study indicated that BR liposome had a greater antiproliferative effect than curcumin liposome, within the optimum time of 24 hours.

**Flow cytometry** **analysis of apoptotic cancer cells**

**Fig. 5:**
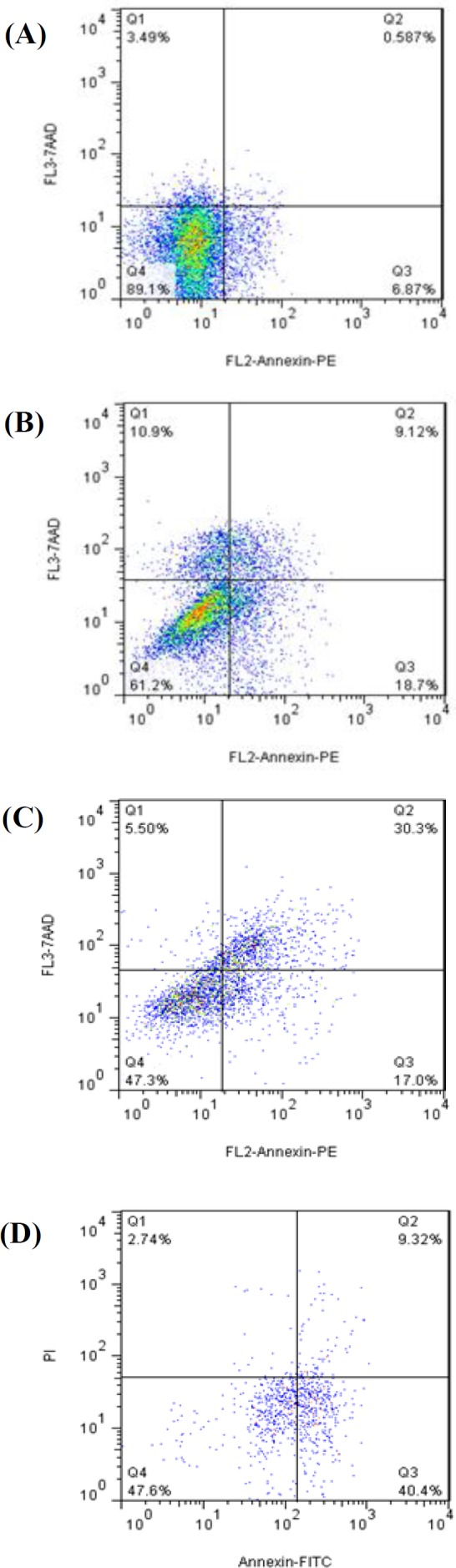
Flow cytometry analysis of apoptotic cancer cells. (A) untreated cells, (B) QU-DB cells treated by curcumin liposome, (C) bromocriptine liposome, and (D) curcumin and bromo-criptine liposomes

The maximum number of apoptotic cells (27.82%) was detected at the concentration of 20 µM for curcumin liposomes, 47.30% at the concentration of 25 µM of BR liposome, and 49.72% in their co-administration by reducing the number of necrotic cells. Data are shown in Figure 5.

## DISCUSSION

The delivery of chemotherapy agents to solid tumors and raising their bioavailability have been a key challenge in the recent biomedical research. Medical imaging and targeted drug delivery using nanotechnology-based tools are rapidly growing to repond this challenge^[^^15^^,^^16^^]^.

The liposomal curcumin prompted *in vitro* apoptosis of human pancreatic cells and down-regulated NF-kB machinery. The results of a study on A549/DDP multidrug-resistant human lung adenocarcinoma cells showed that curcumin stimulates apoptosis through a miRNA signaling pathway^[^^17^^]^. Peng *et al.*^[^^18^^] ^have reported increased cell apoptosis via Akt-Bad signaling pathway in U2OS cells by curcumin-loaded nanoparticles. Other researchers have used a combination of 2-hydroxypropyl-γ-cyclodextrin and liposomes to enhance the curcumin bioavailability and aqueous solubility and encapsulation efficiency, in comparison to the sole use of liposome as the drug delivery vehicle. Their results indicated both *in vivo* and *in vitro* anticancer capacity for 2-hydroxypropyl-γ-cyclodextrin/curcumin-liposome complex against KHOS cell line^[^^19^^]^. Koshkina *et al.*^[^^20^^]^ employed 9-nitrocamptothecin liposome aerosol against osteo-sarcoma lung metastases in mice. Their results showed a significant decrease in the number of tumor foci and the size of tumor nodules in the lung. An early investigation has shown the usefulness of dopaminergic agonists in treatment of lung cancer^[^^21^^]^. BR acts mainly via D2 receptors, through binding to to adenylyl cyclase and reducing intracellular cAMP. By suppressing the cAMP levels, peptide secretion would be inhibited in a dose-dependent manner ^[^^22^^]^. More studies have investigated the effects of dopamine neurotransmitter agonists on cancer cells^[^^23^^,^^24^^]^.

Previous findings have highlighted a quantitatively significant difference for D2-like dopamine receptor genes expression in the NSCLC, among all kinds of dopamine receptor genes^[^^11^^,^^14^^,^^25^^]^. Such significant changes could be used to diagnose, treat, and monitor NSCLC^[^^11^^]^. In supplementary studies, cell proliferation and of D2 receptors expression studies were performed before and after treating cancer cells by BR^[^^11^^,^^14^^,^^25^^]^. It has also been discovered that BR-induced apoptosis in lung carcinoma cells, by activating D2 dopamine receptors and plasma membrane changes, occurs in the early stages of apoptosis^[^^14^^,^^25^^]^. Fadok *et al.*^[^^26^^]^ have found that during the expansion of apoptosis, macrophages specifically recognize PS exposed to the surface of lymphocytes. In this case, phagocytosis of cells and apoptotic bodies are performed, and the organisms are protected from inflammation, leading to the exposure of cellular compositions. Therefore, according to the results of our previous studies, we designed and constructed two nanoliposomes, which efficiently were encapsulated in a polymer structure^[^^11^^,^^14^^]^. This design led to the enhanced aqueous solubility of the mentioned hydrophobic agents and the bioavailability of drugs. 

In this research, anti-cancer potency of nano-formulations without cytotoxicity effects on normal cells was confirmed by co-administration of curcumin and BR nano-liposomes in lung cancer cells. Moreover, BR can be suggested as a valuable agent for use in nano-liposomal drugs for medical management of lung cancer.

## CONFLICT OF INTEREST.

None declared.
